# Semiochemical and Communication Ecology of the Emerald Ash Borer, *Agrilus planipennis* (Coleoptera: Buprestidae)

**DOI:** 10.3390/insects10100323

**Published:** 2019-09-27

**Authors:** Peter Silk, Peter Mayo, Krista Ryall, Lucas Roscoe

**Affiliations:** 1 Natural Resources Canada, Canadian Forest Service—Atlantic Forestry Centre, 1350 Regent Street, Fredericton, NB E3B 5P7, Canada; 2 Natural Resources Canada, Canadian Forest Service—Great Lakes Forestry Centre, 1219 Queen Street East, Sault Ste. Marie, ON P6A 2E5, Canada

**Keywords:** *Agrilus planipennis*, semiochemistry, buprestids, pheromones, host kairomones, chemical ecology

## Abstract

Knowledge of buprestid chemical ecology is sparse but the appearance of the invasive pest *Agrilus planipennis* Fairmaire in North America has provided the impetus to study in detail the semiochemistry and ecology of this important buprestid. The macrocyclic lactone (3Z)-12-dodecenolide [(3Z)-lactone] is identified as a key antennally-active compound that is produced by females and attracts males. Though a weak trap attractant alone, when combined with the host kairomone (3Z)-hexenol and the important visual cue of a green canopy trap, significant increases in male trap capture occur, thus defining (3Z)-lactone as both a sex pheromone of *A. planipennis* as well as the first and only known buprestid pheromone. The non-natural stereoisomer (3E)-12-dodecenolide and the saturated analog, 12-dodecanolide also exhibit mimetic activities towards male *A. planipennis*, suggesting a notable plasticity in this pheromonal structural motif. Efficient synthetic routes to these compounds have been developed. A series of fluoro-12-dodecanolides has also been synthesized containing CF_2_ groups as a strategy to bias the conformational space accessed by these macrolides and to assess if the analogs may act as mimetics for 12-dodecanolide pheromones associated in *A. planipennis*. These compounds also afford a unique opportunity to study the binding affinities of lactone surrogates with *A. planipennis* chemosensory proteins and olfactory receptors. Some progress has also been made in identifying the genes involved in the reception, processing and degradation of volatiles in this invasive insect. It is now evident that the behavior and ecology of *A. planipennis* involves a complex pattern of sensory modalities, including visual, tactile, olfactory and potentially acoustic components. Earlier reviews focused on studies of attractive host volatiles in development of a trapping system for early detection and visual and contact phenomena in *A. planipennis* mate finding. This review will update the semiochemistry and chemical ecology of *A. planipennis* and discuss studies on chemistry and behavior that have identified female-produced pheromone components and host kairomones.

## 1. Background

*Agrilus planipennis* Fairmaire (Coleoptera: Buprestidae) (EAB) is an invasive species originally from Asia that was first detected in North America in 2002 [1]. Though not a major pest in its native range of China, Japan and the Russian Far East, EAB has caused significant and widespread mortality within both planted and natural populations of *Fraxinus* sp. (ash) (Oleaceae) in both Canada and the United States. At the time of the discovery in North America, little was known about the host- and/or mate-finding behavior of EAB. Indeed, very little information regarding the chemical ecology of any buprestids existed. Some evidence demonstrating the importance of visual cues in mate finding was observed by Gwynne & Rentz [2], while the potential of female-derived pheromones was suggested by Dunn & Potter [3]. Unlike other large coleopteran families such as Cerambycidae and Scarabaeidae, an understanding of the mechanisms by which insects locate conspecifics and orient themselves within complex environments was lacking.

The early literature on the mating behavior of buprestids [2,4,5,6,7] described the involvement of visual and tactile cues for mate location. Host tree location has been described as being mediated first by olfactory processes and then mate location by visual or by vibratory, tactile cues or both [4]. Evidence of a pheromone-based mate location mechanism was suggested in experiments with the related two-lined chestnut borer *A. bilineatus* (Weber), where males were observed to significantly prefer cages containing females on host logs over cages containing logs alone [3]. Only recently has additional progress been made into elucidating the pheromone chemistry of buprestids in the form of the extensive research conducted on EAB. The presence of a contact pheromone was suggested [7], which was subsequently identified on the female as 9-methylpentacosane [8]. This compound appears only on the female cuticle of EAB at sexual maturity and stimulates full copulatory activity in males upon antennal contact. A volatile, antennally-active and predominantly female-produced, macrocyclic lactone, (3Z)-12-dodecenolide [(3Z)-lactone], was also described [9] and while identified as the first putative volatile pheromone structure ascribed for EAB, no behavioral activity was reported. It was also demonstrated in laboratory sensory deprivation experiments [10] that male EAB were able to locate and identify females at close range using olfaction pointing to an unidentified volatile cue.

It is now evident that the behavior and ecology of EAB involves a complex pattern of sensory modalities, including visual [11], olfactory [12,13,14], tactile [8,15] and possibly acoustic cues [16]. These host and conspecific interactions blend to influence a variety of host- and mate-location behaviors that are difficult to deconvolute or, indeed, observe independently. Knowledge of the chemical ecology of this species is important to aid in developing sensitive baited traps and other means for early detection at very low insect densities. This review builds on two previous articles [14,17] summarizing and updating the chemical ecology of EAB.

## 2. Visual Cues

Visual cues have long been thought to be critical to mate finding in the Buprestidae [2,18]. Several studies demonstrated that male EAB would land on, and attempt to mate with, dead EAB adults pinned to the foliage of host ash trees [7,8,19,20,21]. Though important in demonstrating the significance of visual cues within the mate location process, using dead (or dummy) females in any assay removes any possible role of the live female (e.g., movement, pheromone emission) from the behavioral equation, thus potentially biasing the results. Similar mate location behaviors that are mediated by visual cues have been reported for other *Agrilus* [22], including the native ash borer *A. subcinctus* Gory and *A. cyanescens* Ratzeburg. In addition to conspecifics, individual males of both species would land on other insect species that were approximate in size and color to their own [22]. Similarly, it was reported [23] that adult male two spotted oak borer *A. biguttatus* (Fabricius), the European oak borer *A. sulcicollis* Lacordaire and *A. angustulus* (Illiger) tend to land on both dead female models of their own as well as decoys of closely related species [24]. These results strongly indicate that visual cues are essential for mate location and subsequent attempted mating by adult male *Agrilus* beetles. The integration of these cues in detection strategies was subsequently investigated. Males were captured on sticky traps made from ash leaves with a dead EAB pinned to the leaves or on blue or yellow sticky cards, with dead EAB attached to them [25]. An interaction between color and presence of dummy beetles was evident in visual attraction by *A. cyanescens* [25]. More males were captured on blue (versus yellow) sticky card traps but only blue traps to which dead beetles had been affixed. No studies have attempted to determine the relative importance of these various visual cues versus other cues associated with mate location.

## 3. Host Kairomones

Considerable research has been conducted into identifying host volatiles that are attractive to EAB since its discovery in North America [26]. Crook & Mastro [17] recently reviewed the body of knowledge concerning the chemical ecology of this species, in which they discussed aspects of trap design, color and host volatiles that are utilized within an effective trapping system. Two main sources of host volatiles were recognized: bark sesquiterpenes [12,17,27,28] and foliar volatiles [13,14,17,27,29]

### 3.1. Bark Sesquiterpenes

Artificially stressing or girdling ash trees is a useful methodology to study attraction to hosts by adult beetles. For example, the two-lined chestnut borer *A. bilineatus* (Weber) landed on girdled host trees (white oak, *Quercus alba* Linnaeus (Fagaceae)) but not on girdled non-host tree genera [30], signaling the production of attractive volatile cues specific to *Q. alba*. Similarly, increased adult male EAB captures as well as significantly higher larval densities were observed on girdled *Fraxinus* sp. [31,32] versus untreated or wounded trees, those treated with herbicide and those exposed to methyl jasmonate [33]. Increased captures of adults following girdling of host trees is hypothesized to be due to changes in volatile emissions, signaling stress in the trees. Changes in ash host volatiles following girdling have been evaluated in several studies [6,12,17] showing increased captures of adults [31,32,33]. Analysis of the volatile blend generated by girdling identified several antennally-active sesquiterpenes, including—α-cubebene, α-copaene, 7-*epi*-sesquithujene (7-EST), trans-β-caryophyllene and α-humulene [12,28]; a sixth antennally-active compound was later identified [34] as the sesquiterpene, eremophilene. Sesquiterpenes are often difficult to synthesize; however, blends of these sesquiterpenes can be found in essential oil distillates, including Manuka oil (a steam distillate from *Leptospernum scoparium* Forst (Myrtaceae)) and Phoebe oil (an essential oil from *Phoebe porosa* (Nees and Martius) Mez (Lauraceae)) [12]. Given the availability of these distillates, field studies that investigated the applicability of sesquiterpene blends within suitable trapping systems utilized these oils as the primary olfactory stimuli [13,17,27,35,36]. Higher trap captures and detection rates on purple traps baited with Manuka or Phoebe oil have been reported [36]. Adults were also attracted to live trees versus girdled trees with sticky band traps baited with Manuka oil, whether visible or hidden from view, indicating the importance of other specific chemical cues being used in orientation to host ash trees in EAB [10]. Healthy host ash trees may emit an entirely different array of volatile compounds. Thus, additional research into volatile chemistry in apparently healthy ash trees and in those trees initially preferred by EAB for attack, may be necessary to fully identify specific compounds and blends that are used to elicit primary attraction and impart host specificity.

### 3.2. Foliar Volatiles

In addition to the analysis of bark sesquiterpenes, detailed research into the foliage volatiles of white and green ash trees (*F. americana* Linnaeus and *F. pennsylvanica* Marshall, respectively) has also been conducted [37], with (3Z)-hexenol and (3Z)-hexenyl acetate comprising > 80% of the foliar volatiles emitted by the two species. Male antennae are highly responsive electrophysiologically to (3Z)-hexenol, compared with any of the other seven green leaf volatile (GLV) compounds [37]. Attraction to (3Z)-hexenol was subsequently demonstrated when it was used as bait on light or dark green sticky traps [13,27,38] or on purple sticky traps [37]. Increased capture of males on traps baited with (3Z)-hexenol compared with unbaited traps or those baited with blends of GLVs were established [35]. Interestingly, the addition of (2E)-hexenol, (2E)-hexenal and hexanal, to traps baited with (3Z)-hexenol reduced trap captures of males compared with those baited with (3Z)-hexenol alone [27,35]. In other tests, (3Z)-hexenol increased captures but not always significantly, on dark green traps [29]. The addition of Manuka oil to traps baited with (3Z)-hexenol did not further enhance captures [27]. Increased capture of males in traps baited with (3Z)-hexenol, as opposed to sesquiterpenes (using Phoebe oil as surrogate) was clearly demonstrated [35] It is not clear, however, if this specific compound is eliciting primary attraction to the host ash trees. Age and reproductive maturity of the insect may influence attraction of EAB to different host volatiles. For example, females may be initially attracted to the foliar volatiles in order to feed and promote maturation [26] and then, given their requirement for ovipositing within crevices and cracks on the trunk of the host tree, be subsequently attracted to bark volatiles associated with this region of the tree. Females caught on traps baited with (3Z)-hexenol are significantly less mature with respect to stage of egg development than females caught on traps baited with bark sesquiterpenes [39]. This suggests that traps baited with (3Z)-hexenol or bark sesquiterpenes do not attract females when they first emerge because they are reproductively immature [40]. It is, therefore, not clear what compounds are attractive to immature newly emerged females presenting another gap in the knowledge of the chemical ecology of this species. Despite considerable research effort, it remains surprising that specific ash-host compounds eliciting primary attraction in adult EAB are still poorly described.

## 4. The Natural Female-Produced Sex Pheromone

**Figure 1:**
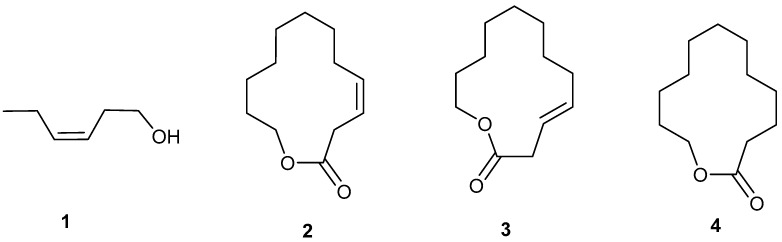
Green Leaf Alcohol and Macrolide EAB pheromones.

Analysis by gas chromatography/mass spectrometry (GC/MS) of volatiles from adult virgin female EAB confirmed [13] the emission of (3Z)-lactone (**2**) [(3Z)-12-dodecenolide] [9] from individual females in the low ng/d range; its geometric isomer, (3E)-lactone (**3**) [(3E)-12-dodecenolide] was not detected (Figure 1); (3Z)-lactone is not an unprecedented chemical structure, being previously reported as the major component of the male-produced pheromone of the cucujid flat grain beetle *Cryptolestes pusillus* (Schönherr) (Coleoptera: Cucujidae) [41].

In field experiments with purple sticky prism traps, the addition of neither lactone isomer to traps containing ash foliar or cortical volatiles (green leaf volatiles or Phoebe oil, respectively) influenced catches. However, on green prism traps, the (3Z)-lactone significantly increased capture of male EAB when traps were deployed in the tree canopy. Captures of males on traps with both (3E)-lactone and (3Z)-hexenol (**1**) or with (3Z)-lactone and (3Z)-hexenol were increased by 45–100%, respectively, compared with traps baited with just (3Z)-hexenol [13]. In olfactometer bioassays, males were significantly attracted to (3E)-lactone but not the (3Z)-lactone or a 60:40 (3E):(3Z) blend. The combination of either (3E)- or (3Z)-lactone with Phoebe oil was not significantly attractive to males in trapping bioassays.

These results demonstrate that adult male EAB are highly attracted to (3Z)-hexenol and the (3Z)-lactone + (3Z)-hexenol combination, on green prism traps. Adding (3*Z*)-lactone to green sticky prism traps with (3*Z*)-hexenol on the south (sunshine side) aspect of the tree canopy consistently increase trap captures and detection rates at low insect densities compared with using (3Z)-hexenol alone [39]. Such detection of populations at low densities is the critical requirement for any effective detection protocol for monitoring invasive insect populations. This bait combination is more attractive than (3*Z*)-hexenol alone, especially when traps are in a competitive deployment (in close proximity to one another). These data were the first to demonstrate the pheromonal activity of (3Z)-12-dodecenolide by increased attraction with a combination of the pheromone and a green leaf volatile in a buprestid species [13,38,39,42].

## 5. Contact Chemoreception

Visual cues are important in orienting EAB males to potential mates [7,25,43]. Lelito et al. [7] coined the term “paratrooper copulation” for the behavior in which hovering EAB males would rapidly descend from heights of 0.3–1.0 m above the foliage directly onto the backs of dead pinned EAB females. Dead and pinned EAB adults of either sex, whether they had been washed in solvent or not, elicited the same number of approaches by feral male EAB; in addition, males spent comparatively more time investigating principally through antennal contact, unwashed females than males or washed females, suggesting the presence of a contact pheromone on the cuticle of female EAB [7].

Analyses by GC/MS of the elytral hydrocarbons from adult male and female EAB revealed a female-specific compound, 9-methylpentacosane (9-Me-C_25_), which appeared on the cuticle only in sexually mature (~10 days old) females. This hydrocarbon was readily synthesized by Wittig reaction of 2-decanone with (n-hexadecyl)-triphenylphosphonium bromide followed by catalytic reduction to yield (racemic) 9-Me-C_25_, which matched the natural compound by gas chromatography/mass spectrometry (retention time and EI mass spectrum). In field bioassays with freeze-killed sexually mature EAB females, feral males spent significantly more time in contact and attempting copulation with unwashed females than with females that had been washed in n-hexane to remove the cuticular lipids. Hexane-washed females to which 9-Me-C_25_ had been re-applied at female equivalent dosages, elicited similar contact time and percentage of time attempting copulation as unwashed females, indicating that 9-methylpentacosane is a contact sex pheromone component of EAB [8]; the potential chirality of this compound has not been investigated

## 6. Acoustics

While research into visual and chemosensory cues related to mate location has been underway for some time, the first evidence of the production of possible acoustic cues generated by adult males and females was reported relatively recently [16].

**Figure 2:**
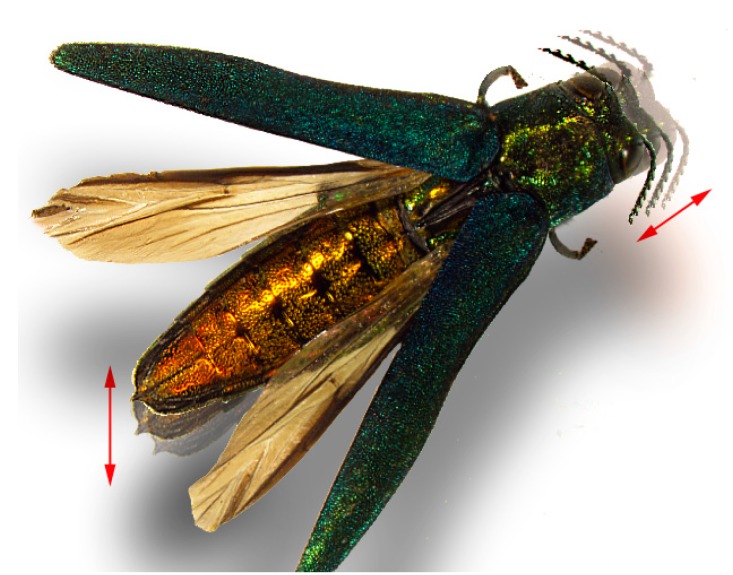
Representation of a male *A. planipennis* undertaking sound-producing behavior (wing opening/head pumping). Arrows indicate direction of movement for both abdomens. (vertically) and head (horizontally) [16].

Simultaneous wing opening and head ‘pumping’ in both males and females produces specific and distinct sounds (Figure 2). Acoustic recordings demonstrate that these sounds consist of a phrase comprising a ‘click’ produced by wing opening, followed by two separate chirps corresponding to the outward and subsequent inward movement of the head with no significant differences in sound parameters between males and females. It was observed that the proportion of males and females exhibiting these specific behaviors were significantly greater under both halogen and incandescent light than in darkness, signifying the importance of the spectral characteristics of the light source on the production of acoustic signals. Although no evidence of these behaviors being related to the courtship sequence were found under these conditions, the results indicate a sound-generation behavior in EAB that is significantly influenced by light conditions. Scanning electron microscopy images indicate the presence of *pars stridens* and plectrum structures on the ventral surfaces of the pronotum and gula and in both males and females. The hearing apparatus and its function in EAB has not been explored but research into this and the total acoustic phenomena could yield enlightening results [44].

Observation and recordings of acoustic phenomena with EAB in natural surroundings have not been made so the possible role of this behavior in natural surroundings is unknown and further research is warranted.

## 7. Progress in Identifying Odor Processing Genes in EAB

The female-biased expression of candidate odorant reception genes will help us to understand the host- and courtship-cues deployed by female EAB. Homology modeling and molecular docking of the predicted odor reception proteins in combination with RNA interference (RNAi) experiments may also reveal critical molecular targets of compounds known to be active toward EAB (e.g., host green leaf volatile, (3Z)-hexenol or indeed the sex pheromone, (3Z)-lactone). Transcriptomic analysis of EAB antennae has revealed the expression of genes involved in volatile odorant perception and signal transduction, including odorant binding proteins (OBPs), chemosensory proteins (CSPs) and olfactory receptors (ORs), notably the obligatory olfactory co-receptor ORCO [45]. Current work is underway to obtain a more detailed picture of sex-specific differences in antennal gene expression, by using the sequencing-by-synthesis technology (i.e., Illumina sequencing). In vitro assays measuring the affinity of ash volatile odorants to recombinantly expressed EAB CSPs are also being performed (D. Doucet, pers. comm.).

Continued studies should include work on host compounds (e.g., (3Z)-hexenol and 7-*epi*-sesquithujene) but also the affinity of the female-produced sex pheromone (3Z)-12-dodecenolide [13,39], a range of structural analogs, including CF_2_ fluoro-analogs as pheromone mimetics [46] and the contact-active compounds [8,15].

## 8. Semiochemistry of EAB Parasitoids

Research into the potential application of both classical and augmentative biological control agents has been ongoing since shortly after the discovery of EAB in North America [47]. Surveys of natural enemies in China and Russia identified several natural enemies that regularly attack EAB. Of these, four species have been approved for use in the United States—*Spathius agrili* (Yang) (Hymenoptera: Braconidae), *S. galinae* Belokobylskij, *S. floridanus* Ashmead, *Tetrastichus planipennisi* (Yang) (Hymenoptera: Eulophidae) and *Oobius agrili* (Hymenoptera: Encyrtidae) [48]. After rigorous non-target host range testing, permits for release in the United States were granted in 2007 [47]. Since then, thousands of lab-reared parasitoids have been released in both Canada and the United States [48], with establishment of released natural enemies being regularly observed in subsequent seasons after release [49,50,51,52].

The native generalist *S. floridanus* Ashmead (Hymenoptera: Braconidae), has been proposed as a biological control agent of EAB. It is a native, larval idiobiont, ectoparasitoid and its host range includes beetles colonizing at least seven host tree genera. It also attacks *A. anxius*, *A. bilineatus* and EAB [53,54] in contrast with *S. agrili*, which has only been observed to parasitize *Agrilus*, almost exclusively EAB, even under no-choice conditions in the laboratory and is almost exclusively associated with *Fraxinus* in the field.

The development of suitable monitoring tools for measuring parasitoid populations following releases is a desirable component for evaluating the effectiveness of releases. While several methods for detecting parasitoids exist, in only one instance has a semiochemical-based detection system been examined. For *S. agrili*, seven male-specific volatile compounds have been identified and synthesized of which, three of these, dodecanal, (4R,11E)-4-tetradecenolide and (Z)-10-heptadecen-2-one, were the key behaviorally active components in flight tunnel bioassays [55]. Male specificity was demonstrated by GC analysis of male and female volatile emissions and whole body extracts. Both the racemic and chiral forms of the γ-lactone and both E- and Z-isomers, were synthesized. Flight tunnel tests showed positive male and female *S. agrili* responses to both natural pheromone and synthetic blends, with upwind flight and landing on the source. Field-cage studies with yellow sticky-card traps also indicated that both adult males and females were caught in significantly higher numbers on traps baited with the male pheromone than on unbaited traps. An effect of mating on female chemotaxis was also observed, with unmated females displaying upwind flight to the pheromone source, while mated females did not. A female pheromone blend was also identified but its behavioral role could not be determined. While this technology is now available, its possible use is secondary to the utilization of yellow pan traps to measure parasitoid establishment in releases sites [56].

Investigations into the role of host-plant synomones in the host location has been undertaken for *S. floridanus* as well as for *S. agrili*. In laboratory assays, it has been demonstrated [54] that *S. agrili* are attracted to leaf tissue cues whereas *S. floridanus* is attracted only to EAB larvae in stem tissue. Knowledge of host-location behavior may improve biological control by these parasitoids, by supporting approaches for pre- and post-release strategies. Similar to the male-derived pheromones identified for *S. agrili*, these volatile semiochemicals may be useful in future monitoring tools for evaluating *Spathius* sp. populations after release.

In addition to natural enemy surveys in China, collections of natural enemies attacking EAB in North America were also undertaken. These yielded several potential candidates for augmentative biological control, including—*Leluthia astigma* Ashmead, several *Atanycolus* spp., *Spathius floridanus* Ashmead (Hymenoptera: Braconidae), *Phasgonophora sulcata* Westwood (Hymenoptera: Chalcididae) and *Balcha indica* Mani and Kaul (Hymenoptera: Eupelmidae) [57]. In only one species, *P. sulcata*, has evidence of a pheromone been observed. Roscoe et al. [58] described the mating sequence of this species and determined, using laboratory bioassays, that females produced a volatile pheromone that elicited upwind walking and distinct behaviors in conspecific adult males. While a pheromone has not yet been identified, the significant number of males attracted to upwind females suggests the potential of its utilization in detection traps for monitoring *P. sulcata* populations. Evidence of female attraction to volatile cues of both adult EAB and host tree volatiles was also observed. It was also observed that females preferentially chose (3Z)-12-dodecenolide over a control odor source [59], as well as a green-leaf volatile blend from infested *Fraxinus* sp. over clean air in laboratory bioassays. While this suggests the importance of these odor sources in a potential monitoring program, field evaluation of these semiochemicals in conjunction with a suitable trapping system has not yet occurred.

## 9. Development of EAB Detection Tools

### 9.1. Trap Development

The appearance of EAB in North America and its devastating ecological and economic impacts have created an opportunity to study the semiochemistry, biology and ecology of this buprestid in detail [14]. To reiterate, adding (3Z)-lactone to green sticky prism traps baited with (3Z)-hexenol, consistently shows highest captures and detection rates at low insect densities on the south aspect of the tree canopy [38,39]. This is the trap option that is recommended for early detection of EAB in Canada and elsewhere [60] together with branch sampling for delimitation surveys [61]. Multi-funnel green traps have also been developed with fluon^®^ coating improving trap capture [62]. Further work should be carried out to estimate the required trap densities, including establishing the “active space” (*sensu* Cardé & Baker [63]) of the traps [64] in order to help refine this technique in particular with risk-based models.

### 9.2. Effective Range of the Trap

For modelling the range of attraction of the pheromone, (3Z)-lactone, some recent data has been collected [64] to improve the understanding of the effectiveness of green sticky prism traps baited with host kairomone and insect pheromone lures for EAB. Traps were deployed over a single flight season in urban locations of Ontario, Canada with relatively low densities of emerald ash borer (ca. 5 galleries/m^2^ of outer bark). Traps were placed in pairs of trees separated by not more than 25 m. All traps contained the host kairomone, (3Z)-hexenol, with the remaining half in each pairing additionally baited with (3Z)-lactone pheromone. Both lure types were highly effective in capturing EAB, with >90% detection rates overall at these insect densities. However, traps baited with the pheromone and host volatile lures doubled trap captures of EAB over distances of at least 25 m from the nearest traps baited with only host volatiles. Although the baseline detection rate of traps containing (3Z)-hexenol alone is not significantly reduced compared with traps containing (3Z)-lactone, the overall trap effectiveness is significantly increased when (3Z)-lactone is present. The implications for the use of (3Z)-lactone in an early-warning trapping system are discussed. Trap layout methods and risk-based analysis models can now be further refined by including these data about the attractive range of lures and their effectiveness in different plot environments.

### 9.3. Mechanism of Pheromonal Activity

Our previous studies have shown: Firstly, (3Z)-lactone is a weak attractant alone but augments trap capture combined with the host volatile (3Z)-hexenol [38] and secondly, it does not elicit (walking) anemotaxis to a (3Z)-lactone source in an olfactometer assay whereas, curiously, (3E)-lactone does [13]. Thirdly, (3Z)-lactone lures (×6) surrounding a (3Z)-lactone + (3Z)-hexenol baited trap do not shut down trap capture. Indeed, trap capture increases significantly and the more trap-disruptors that are placed around the center trap, the higher the trap capture (unpublished data).

Although the mechanisms involved are likely complex, we hypothesize that the mode of action of the natural lactone with EAB is in part, therefore, more as a “flight arrestant” and not just an elicitor of classical chemoanemotaxis. The kairomone, (3Z)-hexenol, for example, would bring males toward the host and the pheromone guides males to females in the tree by arresting flight activity and beginning their search behavior [10]. This may be guided by a combination of visual, acoustic and contact chemoreception as well as olfactory cues. This hypothesis remains to be fully tested. Trap capture per se is a very complex and perhaps poor proxy for understanding insect behavior, especially mate location and mechanistic arguments gleaned from this must be viewed with caution.

## 10. Attractants for Other *Agrilus* Species

The only other *Agrilus* species, to our knowledge, where putative kairomones have been examined, is the two-spotted oak buprestid, *A. biguttatus* (Fabricius) (Coleoptera: Buprestidae), a species native to Europe that attacks oak trees (*Quercus* spp.) and is a high invasive risk to North America. Biologically active compounds from oak foliage and bark were tested by GC/EAD and GC-MS and several synthetic blends, which evoked strong positive behavioral responses in olfactometer tests, were identified [65]. These comprised green leaf volatiles and terpenoids but have not yet been tested in trapping studies.

Girdling of host trees (*Betula papyrifera* Marsh) elicits significant attraction of adults of the bronze birch borer *Agrilus anxius* Gory (Coleoptera: Buprestidae) to purple prism traps and can be used as a preliminary detection tool for this species [66]. Trap color is also an important visual cue in host location by this species, with unbaited green prism traps showing higher trap capture rates than purple or white prism traps. Rutledge also reported [67] that green multi-funnel traps hung on girdled birch trees are effective in trapping *A. anxius*. The highly antennally-active spiroketal—conophthorin—produced by the birches *Betula papyrifera* [66] and *B. pendula* Roth. (*Betula* spp., Fagales: Betulaceae) [68], does not appear active in traps alone or combined with (3Z)-hexenol [66]. Further research is required to determine any pheromone component of a lure for *A. anxius*. This is especially important since many kairomones and indeed pheromones, may be inactive unless presented together in traps [69,70,71]. Antennally-active host volatiles or those of host-fungal origin may also be important kairomones for *A. anxius*. Due to the high susceptibility of European *Betula* spp. to *A. anxius*, concern has been raised about its possible introduction into Europe, the UK and Asia [72] where if established it could cause widespread birch mortality on a continental scale.

## 11. Pheromone Analogs

### 11.1. Macrocycle Isomers

The female-produced sex pheromone of EAB has been shown to comprise the macrocyclic lactone, (3Z)-12-dodecenolide **2**. This compound and its geometrical isomer analog, (3E)-12-dodecenolide **3**, have been demonstrated previously to stimulate electrophysiological responses and, in combination with a host-derived green leaf volatile, (3Z)-hexenol **1**, to be attractive (effect trap capture) to male EAB in green prism traps deployed in the ash tree canopy. It has also been shown that the saturated analog, 12-dodecanolide **4**, is similarly active, eliciting a significant antennal response and significant attraction of EAB in both olfactometer and trapping bioassays in green traps with (3Z)-hexenol [73].

Conformational modeling of these three lactones [73] reveals that their energies and shapes are very similar, suggesting they might share a common receptor in EAB antennae; activity of all three also suggests that the critical pharmacophores are the lactone entity and the conformation of the ring. These findings provide new insight into the pheromone ecology of this species, highlighting the apparent plasticity in response of adults to the pheromone and its saturated analog prompting further study. The saturated analog can be made cheaply, in high yield and on a large scale via Mitsunobu esterification of a saturated ω-hydroxy acid or, more simply, by Baeyer-Villiger oxidation of commercially available cyclododecanone. The analog can thus provide an inexpensive option as a lure for detection surveys as well as for possible mitigation purposes, such as mating disruption or push-pull strategies [60].

### 11.2. Macrocyclic Fluoro Analogs

**Figure 3:**
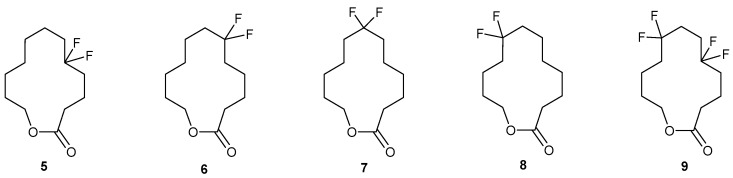
Fluorinated 12-dodecanolides.

Fluorine has advantages as a hydrogen mimetic for biologically-active molecules in that it is sterically compact and does not enter into strong intermolecular interactions [74]. It has been established that the CF_2_ group tends to occupy corner positions when it is incorporated into aliphatic rings; in particular, medium sized macrocyclic compounds [75]. The larger size of fluorine over hydrogen also avoids transannular interactions relative to hydrogen, while the electronegativity of fluorine changes the hybridization towards sp^2^ at the directly bound carbon widening the C-CF_2_-C angle [76]. Hence, five 12-dodecanolides (Figure 3) were synthesized containing CF_2_ groups at C5, C6, C7, C8 and in one case a double substitution at C5 & C8, as a strategy to bias the conformational space accessed by these macrocycles and to determine if the analogs act as mimetics for 12-dodecanolide pheromones associated with the EAB [46]. The CF_2_ group would place conformational constraints at different locations into these molecules, as an alternative strategy to introducing double bonds of defined geometry such as is found in **2** and **3**. Thus, syntheses of 5,5-difluoro-**5**, 6,6-difluoro-**6**, 7,7-difluoro-**7**, 8,8-difluoro-**8** and 5,5,8,8-tetrafluoro-**9**, 12-dodecanolides were carried out.

**Figure 4:**
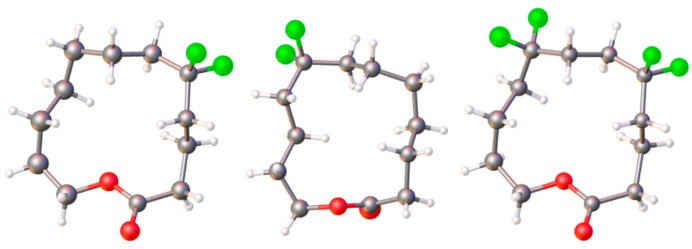
Representations of the X-ray derived structure of 12-dodecanolides **5**, **8** and **9** (left to right). In all cases the CF_2_ groups adopt corner locations in the solid state.

X-ray structural data was obtained for three (**5**, **8** and **9**) of these compounds [46]. The structures show that the CF_2_ groups indeed do occupy ‘corner’ positions in the macrocycle consistent with their ability to bias accessible conformations (Figure 4). The fluorine containing 12-dodecanolides all generated an electro-antennogram response in female beetles and ongoing work shows that several effect trap capture as (3Z)-lactone mimetics (unpublished data).

## 12. Synthetic Chemistry of EAB Macrolides

The syntheses employed for (3Z)-12-dodecenolide **2** (Section 12.1) and (3E)-12-dodecenolide **3** (Section 12.2) were very similar; a lithium salt-free Wittig reaction was used to install the *Z*-olefin in the twelve carbon chain of **2** and a Julia-Kocienski olefination was used to install the *E*-olefin in **3**. In both of these olefinations, a 3-carbon fragment was added to the aldehyde **12** to increase the chain length from 9 to 12 carbons and the lactone functionality was installed in the same way for both **2** and **3**. Both of these syntheses involved 6 steps and were adapted from the synthesis of **2** reported by Boden et al. [77] and used the same basic pathway; the only addition was of a protecting group (EVE, ethyl vinyl ether) on aldehyde **12**. In an effort to shorten the synthetic pathway to **2**, a 3-step procedure was devised; see Section 12.3. Also, a 1-step synthesis of the saturated lactone analog **4** was implemented (Section 12.4).

### 12.1. Synthesis of (3Z)-12-Dodecenolide 2

**Scheme 1:**
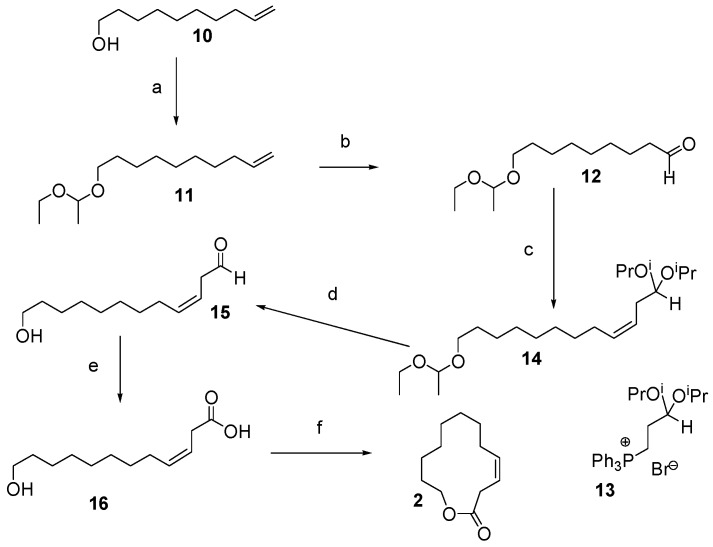
Synthesis of (3Z)-12-dodecenolide **2**.

The macrocyclic lactone, (3Z)-12-dodecenolide **2** ((3Z)-lactone, Scheme 1), was synthesized according to the procedure described by Boden et al. [77] and used by Bartelt et al. [9] with the addition of an ethyl vinyl ether (EVE) protecting group introduced to 9-decen-1-ol **10** (which doubled the yield of the subsequent Wittig step) [13,78]. After EVE protection to give **11**, the EVE-protected alkenol **11** was subjected to ozonolysis with reductive workup to give protected hydroxyaldehyde **12** and then Wittig reaction with a Wittig salt containing a protected aldehyde (**13**), gave alkene **14**. Hydrolysis of both of the acetals in **14** to give a (3Z)-unsaturated hydroxyaldehyde **15**, then Lindgren oxidation to a hydroxycarboxylic acid (**16**) and finally a Mitsunobu esterification to effect the macrolactonisation, gave *Z*-lactone **2** in 37% overall yield (6 steps). The synthesis of (3Z)-12-dodecenolide **2** was, therefore, successfully accomplished with the IR spectra, EI (70 eV) mass spectra and ^1^H and ^13^C NMR spectra closely matching those previously reported [77]. Formation of (3*E*)-12-dodecenolide **3** was found to be intrinsic to the synthesis at approximately 2%. **3** Could not be separated from **2**. ^1^H NMR supported the presence of ca. 2% of (3*E*)-lactone **3** in the product.

Ethyl vinyl ether (EVE), pyridinium *p*-toluenesulfonate (PPTS), CH_2_Cl_2_, RT, 98%.

- 1. O_3_, CH_2_Cl_2_, −78 °C. 2. PPh_3_, −78 °C—RT, 93%.
- **13** + Sodium bis(trimethylsilyl)amide, tetrahydrofuran (THF) and toluene, 0 °C—RT, then **12**, −99 °C—RT, 90% yield, 98% *Z*-selectivity.
- TsOH, wet THF, reflux.
- NaClO_2_, H_2_NSO_3_H, 1-methyl-1-cyclohexene, CH_2_Cl_2_, H_2_O, 0 °C—RT, 70% over 2 steps.
- diisopropylazodicarboxylate (DIAD) or diethylazodicarboxylate (DEAD), PPh_3_, toluene, RT, 64% with DIAD; overall yield = 37% over 6 steps.

### 12.2. Synthesis of (3E)-12-Dodecenolide **3**

**Scheme 2:**
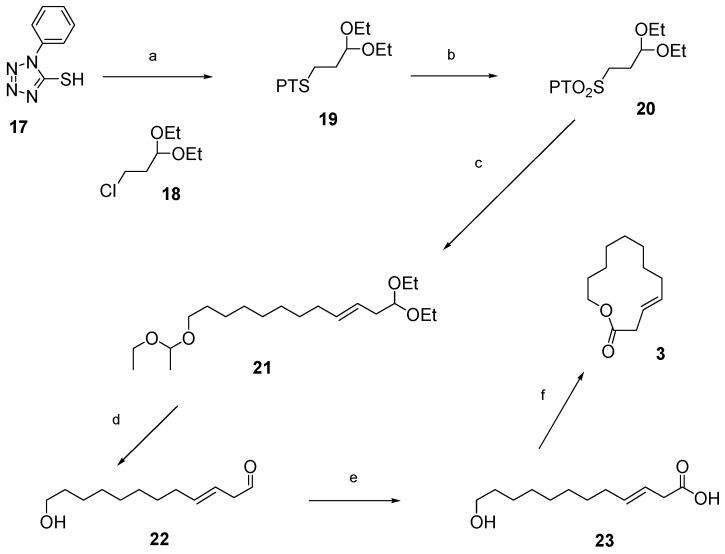
Synthesis of (3*E*)-12-dodecenolide **3** in 97% yield.

The (3E)-lactone [(3E)-12-dodecenolide] (**3**) (Scheme 2) synthesis was successfully accomplished by a Julia-Kocienski olefination according to the methodology previously described [79]. The Julia-Kocienski olefination of aldehyde **12** proceeded with 60% yield and ca. 97% *E*-stereochemistry to give olefin **21**. The phenyltetrazole (PT) sulfone **20** was synthesized by deprotonating 1-phenyl-1H-tetrazole-5-thiol (PTSH) **17** with sodium hydride and coupling it with commercially available **18** to give thioether **19**. Ammonium molybdate tetrahydrate/hydrogen peroxide oxidation of **19** furnished the PT sulfone **20**. After the Julia-Kocienski olefination, double hydrolysis of the two acetals of **21** gave **22** and Lindgren oxidation of **22** gave the 3*E*-hydroxyacid **23**. Finally, as reported by Reference [77], activation of the hydroxyl group using the modified Mitsunobu method [80] gave (3E)-12-dodecenolide **3** in an overall yield of 16% from **17**. The detailed synthesis of (3E)-lactone **3** is described elsewhere [81].

Schlosser modification [82] of the Wittig reaction was initially employed in an attempt to make **21** starting from Wittig salt **13** and aldehyde **12**, however, the *E*-selectivity of the reaction was very capricious, with 80% stereochemical purity being the best result out of a dozen attempts at the reaction. This was deemed to be unacceptable and the much better ~97% stereochemical purity with the Julia-Kocienski olefination which gave **21** was more satisfactory.

- 1. NaH, DMF, 0 °C–90 °C. 2. 3-chloropropionaldehyde diethylacetal **18**, NaI, 90 °C, 76%.
- (NH_4_)_6_Mo_7_O_24_·4H_2_O, H_2_O_2_, EtOH, RT, 73%.
- 1. Potassium bis(trimethylsilyl)amide, dimethyoxyethane (DME), −56 °C, then **12**, −56 °C—RT, 60% yield, 97% *E*-selectivity.
- TsOH, wet THF, reflux.
- NaClO_2_, H_2_NSO_3_H, 1-methyl-1-cyclohexene, CH_2_Cl_2_, H_2_O, 0 °C—RT, 60% over 2 steps.
- DIAD or DEAD, PPh_3_, toluene, RT, 82% with DIAD; overall yield = 16% over 6 steps.

### 12.3. Synthesis of (3Z)- and (3E)-12-Dodecenolide Mixture (**2** and **3**)

**Scheme 3:**
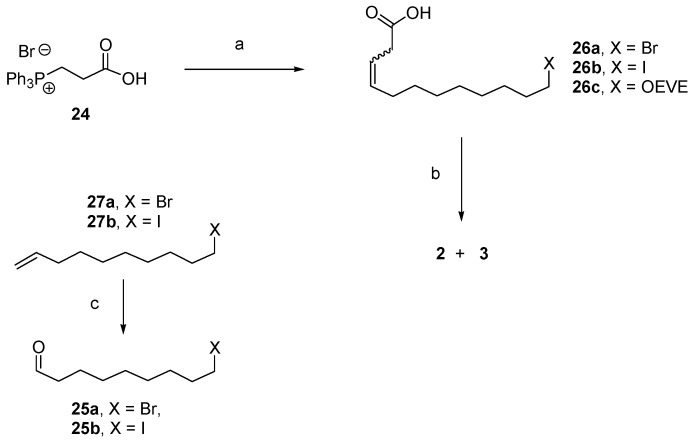
Synthesis of a mixture of 2:1 Z-:*E*-lactone (**2**:**3**).

**Figure 5:**
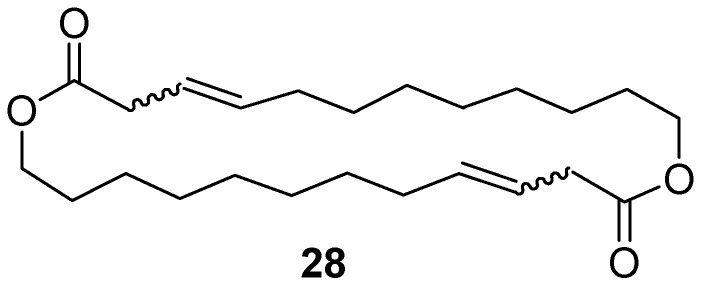
Lactone dimer intrinsic to the synthesis of **2** and/or **3**.

The focus of this synthesis was to use a Wittig reaction to give the 12-carbon chain of **2** with an olefin at position 3 but more concisely than the 6-step procedure shown in Scheme 1. To this end, a Wittig reaction of aldehydes **25a**, **25b** or **12**, with yields of 70%, 67% or 61%, respectively, were obtained using **24** as the Wittig salt, THF as the solvent and 2 equivalents of sodium bis(trimethylsilyl)amide as the base, with a 2:1 *Z*/*E* stereoisomeric ratio in the product **26** for all 3 cases (Section 12.3, Scheme 3). A straightforward intramolecular S_N_2 of **26a** using potassium carbonate (K_2_CO_3_) as base, potassium iodide (KI) as catalyst and refluxing acetone as solvent yielded a 2:1 mixture of **2**:**3** in good yield (82%); **26b** cyclized in the same fashion as **26a**, to a mixture of **2** and **3** in similar yield (72%) and *Z*:*E* selectivity (2:1) but without the KI catalyst. Of note in the synthesis of these lactones, either *Z*-, *E*- or *Z*-,*E*-mixture, is the formation of the dimer **28** (see Figure 5) during the last step (either Mitsunobu cyclization or S_N_2 ring closure) at a level of ~5–10% of the product mixture. The lactone dimer **28** can be separated from the monomer lactone product (either **2**, **3** or the **2**/**3** mixture) by silica gel column chromatography.

- 2 equiv. sodium bis(trimethylsilyl)amide, THF, 0 °C—RT, then **25a**, **25b** or **12**, −78 °C—RT, 70%, 67% and 61% yields, respectively and 2:1 *Z*/*E* in all 3 cases.
- For **25a** and **25b**, K_2_CO_3_, KI (only for **25a**), acetone, reflux, 82% and 72% yields, respectively, 2:1 **2**:**3** (*Z*:*E*) for both **26a** and **26b**.
- 1. O_3_, CH_2_Cl_2_, −78 °C. 2. PPh_3_, −78 °C—RT, 100% yield for **25a** and 93% for **25b**.

**Scheme 4:**
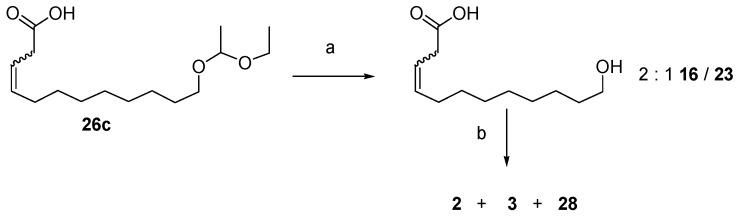
Synthesis of 2:1 *E*:*Z*-lactone **2** and **3** via carboxyacetal **26c**.

Carboxyacetal **26c** could also be obtained in a Wittig reaction employing Wittig salt **24** and aldehyde **12** in similar yield (61%) and *Z*:*E* (2:1) selectivity to acids **26a** and **26b** (Scheme 3). The hydroxyl group of **26c** can then be unmasked with hydrochloric acid in wet THF (quantitative yield) to give a 2:1 mixture of **16** and **23** and the Mitsunobu conditions as previously reported [77] readily formed the lactone as a mixture of *Z*- and *E*-isomers **2** and **3** (84% combined yield), as well as a small amount (~5%) of the dimer **28** (see Scheme 4).

- HCl, H_2_O, THF, RT, 100%.
- DIAD, PPh_3_, toluene, RT, 84% combined yield of **2** and **3**, ~5% **28**. **2**:**3** = 2:1.

**Scheme 5:**
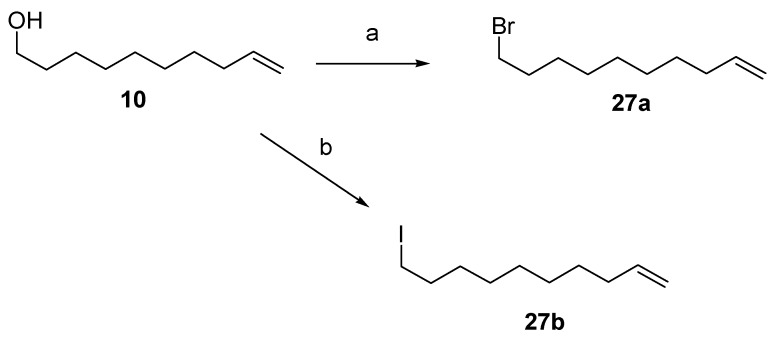
Halogenation of 9-decen-1-ol **10**.

Alkenes **27a** and **27b** are readily prepared in 2 steps from 9-decen-1-ol **10** (see Scheme 5; both yields were quantitative). The commercial availability of **27a** makes **25a** obtainable in only 1 step.

- CBr_4_, PPh_3_, CH_2_Cl_2_, 0 °C, 100%.
- I_2_, PPh_3_, imidazole, 1:3 CH_3_CN/Et_2_O, RT, 100%.

### 12.4. Synthesis of Saturated Lactone Analog, 12-Dodecanolide 4

**Scheme 6:**
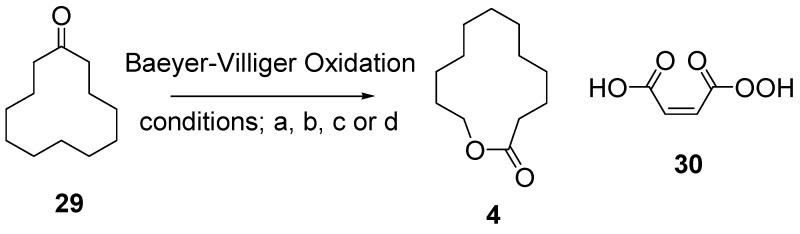
Baeyer-Villiger oxidation of cyclododecanone **29**.

Baeyer-Villiger oxidation (BVO) of cyclododecanone **29** (Sigma-Aldrich, Saint Louis, MO, USA) would yield the saturated lactone analogue **4** in one step (Section 12.4, Scheme 6). Consequently, a straightforward BVO of **29** with *meta*-chloroperoxybenzoic acid (*m*CPBA) would provide a convenient method for the synthesis of **4**. This reaction, however, proved to be sluggish; this was also reported by van der Mee et al. [83] who refluxed **29** and *m*CPBA in CH_2_Cl_2_ for 10 days and still reported incomplete consumption of **29** [73].

Toluenesulfonic acid monohydrate-(TsOH·H_2_O)-catalyzed *m*CPBA BVO [84] of **29** proceeds with 99.8% completion and 87% yield in 3.5 wks. The use of basic conditions (2 equiv. of NaHCO_3_ instead of a catalytic amount of TsOH·H_2_O) gave 98% completion and 59% yield after 2.5 months. Obviously, the latter conditions are somewhat impractical from a synthetic standpoint; however, a large-scale synthesis of **4** from **29** using *m*CPBA and a catalytic amount of TsOH·H_2_O was conducted and **4** was obtained in 87% yield.

The more reactive trifluoroperoxyacetic acid (prepared in situ from trifluoroacetic anhydride and hydrogen peroxide) [85] gave **4** from **29** in 11 d, 72% yield and with complete consumption of the starting material. Also, the reagent Oxone^®^ (potassium peroxymonosulfate, 2KHSO_5_·KHSO_4_·K_2_SO_4_) was completely inert towards **29** when stirred at room temperature in dichloromethane over 2 d and magnesium monoperphthalate hexahydrate (MMPP)/NaHCO_3_ [86] only converted ~0.5% of starting material **29** to product **4** when stirred at room temperature in 1:1 MeOH:H_2_O over 1 d and further stirring at this temperature produced no further conversion.

The reagent permaleic acid (**30**, Scheme 6) is reported to convert **29** to **4** in 1 d [87] and **29** is cleanly converted to **4** by stirring with permaleic acid **30** in CH_2_Cl_2_ at RT for 5 d in 75% yield.

- 2.1 equiv. *m*CPBA, 0.04 equiv. TsOH·H_2_O, CH_2_Cl_2_, RT, 87%.
- 10 equiv. *m*CPBA, 2 equiv. NaHCO_3_, CH_2_Cl_2_, RT, 59%.
- 10 equiv. H_2_O_2_ (35 wt% aqueous), 31 equiv. (CF_3_CO)_2_, 1 equiv. Na_2_HPO_4_·7H_2_O, CH_2_Cl_2_, RT, 72%.
- Permaleic acid **30** (generated in situ from maleic anhydride, 35 wt% aqueous H_2_O_2_ and acetic anhydride), CH_2_Cl_2_, RT, 75%.

## 13. Future Directions

Buprestidae, known as jewel beetles or metallic wood-boring beetles, are among the largest of the beetle families, with ca. 15,500 species known in 775 genera [88]; in the subfamily Agrilina, ~3000 species of *Agrilus* are described. Little is known, however, about the semiochemistry of any buprestids with one notable exception: the emerald ash borer, *Agrilus planipennis* Fairmaire (Coleoptera: Buprestidae) which is detailed in this review.

Thus, (3Z)-12-dodecenolide, the sex pheromone of EAB, remains the only recorded pheromone structure in the Buprestidae at this time [9,13]. Whether or not this macrolide structural motif is conserved in this genus and translates into the chemical ecology of other Buprestidae, is unknown; the lactone motif in chemical communication, however, is a well-known “privileged structure” [89] with the lactone being better suited to serve as a volatile chemical than its hydroxy-fatty acid precursor; cyclisation is entropically favored and the ester can be easily deactivated by hydrolysis. The non-natural stereoisomer (3E)-12-dodecenolide and the saturated analog, 12-dodecanolide and several difluoromethylene-12-dodecanolide analogs exhibit remarkable mimetic activities towards male EAB pointing to noteworthy plasticity in this pheromonal structural motif. These compounds may be useful ligands, as may be host-related kairomones, to study binding to EAB antennal sensory neurons. Indeed, some progress has been made in identifying the genes involved in the reception, processing and degradation of volatiles in this invasive insect.

Successful location of ash trees, mates and oviposition sites by EAB requires the ability to detect chemical and physical cues indicating the presence of these resources within a highly complex environment of a variety of stimuli. The green leaf volatile, (3Z)-hexenol, for example, is ubiquitous in a forest environment, being emitted by almost all hardwood trees. Given its widespread emittance from a variety of angiosperm species in addition to *Fraxinus*, it is interesting that it remains the most effective of host kairomones identified thus far. However, given the lack of EAB recruitment on other angiosperms, it seems reasonable to assume that more additional kairomone components that facilitate orientation to *Fraxinus,* exist. It has been shown [90] that the number of eggs laid by female EAB was significantly lower on healthy trees or “over-infested” (crown class = 5) than on moderately-stressed trees (crown class = 3, 4). This observation seems to indicate variability in host kairomones produced by healthy or over-infested trees, perhaps mitigating female oviposition.

The antennally-active sesquiterpene compounds [12] are weak attractants at best and do not synergize the pheromone [38]. Thus, despite considerable research effort, it remains surprising that specific ash-host compounds eliciting primary attraction in adult EAB are still poorly described and further research is required. A more potent kairomonal “gestalt” blend, if indeed it exists, may have important consequences to the effectiveness of the pheromone at low insect density and its active range. A competitive binding approach using nonlinear responses of olfactory receptors to complex mixtures might be a useful approach [91]. Thus, in vitro binding data with these receptors would provide the opportunity to study the binding affinities of lactone surrogates and host kairomones with EAB antennal receptors (i.e., chemosensory proteins and olfactory receptors).

Insect derived macrolides are now a relatively common structural motif and many are likely biosynthetically derived from fatty acids which serve as starting materials modified by chain-shortening and oxidation processes [89]. Due to the minute amounts available from natural sources they are likely detectable only by mass spectrometry and some general rules for identification have been described [92]. (3Z)-12-Dodecenolide, however, remains the only known pheromone within *Agrilus*. Whether or not this macrolide structural motif is conserved in this genus and translates into the chemical ecology of other buprestidae, is unknown. Hopefully, lessons learned with EAB will aid into unravelling the semiochemistry of other buprestids since it seems certain that many will achieve pest status as world trade practices increase the risk of invasion of these exotic species.
